# Antigenic Characterization of ORF2 and ORF3 Proteins of Hepatitis E Virus (HEV)

**DOI:** 10.3390/v13071385

**Published:** 2021-07-16

**Authors:** Giulia Pezzoni, Lidia Stercoli, Eleonora Pegoiani, Emiliana Brocchi

**Affiliations:** Istituto Zooprofilattico Sperimentale della Lombardia e dell’Emilia Romagna (IZSLER), Via Bianchi 9, 25124 Brescia, Italy; lidia.stercoli@gmail.com (L.S.); eleonora.pegoiani@gmail.com (E.P.); emiliana.brocchi@gmail.com (E.B.)

**Keywords:** HEV, monoclonal antibodies, recombinant antigens, HEV ORF2

## Abstract

To evaluate the antigenic properties of Hepatitis E Virus (HEV) Open Reading Frame 2 and 3 (ORF2 and ORF3) codified proteins, we expressed different portions of ORF2 and the entire ORF3 in *E. coli*, a truncated ORF2, was also expressed in baculovirus. A panel of 37 monoclonal antibodies (MAbs) was raised against ORF2 (1–660 amino acids) and MAbs were mapped and characterized using the ORF2 expressed portions. Selected HEV positive and negative swine sera were used to evaluate ORF2 and ORF3 antigens’ immunogenicity. The MAbs were clustered in six groups identifying six antigenic regions along the ORF2. Only MAbs binding to the sixth ORF2 antigenic region (394–608 aa) were found to compete with HEV positive sera and efficiently catch the recombinant antigen expressed in baculovirus. The ORF2 portion from 394–608 aa demonstrated to include most immunogenic epitopes with 85% of HEV positive swine sera reacting against the region from 461–544 aa. Only 5% of the selected HEV sera reacted against the ORF3 antigen.

## 1. Introduction

Hepatitis E is an important public health infectious disease in developing countries where it is transmitted mainly by the fecal–oral route with a high mortality rate in pregnant women [1]. It also occurs sporadically in numerous industrialized countries where it is considered a food-borne zoonosis [2].

The causative agent of hepatitis E is a “non-enveloped”, single-stranded positive-sense RNA virus (HEV). HEV is classified in the family Hepeviridae that includes two distinct genera: Orthohepevirus (all mammalian and avian HEVs) and Piscihepevirus (fish HEV). The genus Orthohepevirus has been divided into four species, namely Orthohepevirus A–D. Eight main HEV genotypes have been identified within Orthohepevirus A so far, of which genotypes 1–4 and 7 infect humans [3]. While genotypes 1 and 2 infect only humans and are endemic in developing countries, genotypes 3 and 4 have been isolated also in swine and other animal species [4], and are recognized as zoonotic agents responsible for the disease in developed countries. Genotype 7 has been isolated in camels and a human case of chronic hepatitis E after transplantation has been linked to the consumption of food products from camels in the United Arab Emirates [5].

The genome of HEV, approximately 7.5 kb in length, is organized in three partially overlapping Open Reading Frames (ORFs): ORF1 encodes for a non-structural polyprotein, ORF2 encodes for the 660 amino acids’ (aa) long capsid protein, and the ORF3 codes for a small phosphorylated protein whose function is mostly unknown, although it might be involved in virion release from infected cells [6].

The ORF2 encoded protein is responsible for the main humoral immune response and the antigenic properties of ORF2 from typically human genotypes were extensively studied [7,8,9,10]. Antigenic and structural studies on the HEV ORF2 protein were mainly conducted using recombinant proteins expressed in various systems [11,12,13,14,15,16]. In particular, the carboxy-terminal portion of ORF2 (394–660 aa) contains immune-dominant epitopes [12] strongly reactive with both acute and convalescent-phase human sera [11]. Even shorter portions of ORF2 viral capsid such as those named E2 (394–606 aa) and E2s (459–606 aa) display major neutralizing epitopes [17]. Using monoclonal antibodies, neutralizing conformation-dependent epitopes within fragments 452–617 aa [18] and E2s [9,16] of ORF2 were described, while two neutralizing linear epitopes mapped between amino acids 578–607 were isolated by phage display library [19].

Immunogenic properties of the ORF3 protein have been also investigated by a few researchers [7,9,16]; immunization with bacterially expressed ORF3 partially prevented experimental hepatitis in primates challenged with high doses of HEV genotype 1 and 4 [20].

Moreover, some commercial HEV ELISA assays designed for humans include ORF3 peptides as antigens.

In the present study, the antigenic properties of ORF2 and ORF3 proteins from HEV genotype III of porcine origin were studied. The immunological characteristics of ORF2 were evaluated using both monoclonal antibodies (MAbs) and swine sera on a series of recombinant antigens corresponding to the entire capsid protein and its various portions produced in different expression systems, while the immunogenic properties of a recombinant ORF3 expressed in *E. coli* were evaluated using selected swine sera.

## 2. Materials and Methods

### 2.1. Extraction and Amplification of HEV Viral RNA

Viral RNA was extracted from a sample of feces as previously described [21]; the ORF2/ORF3 region (GenBank accession number KF891380) of the genomic HEV RNA was retro-transcribed and amplified by an RT-NESTED-PCR protocol. The amplified DNA was cloned into the vector pCR^®^-Blunt by the Zero Blunt PCR Cloning Kit (Life Technologies, Carlsbad, CA, USA) and sequenced.

### 2.2. Production of Recombinant Antigens

A series of recombinant proteins encompassing the entire ORF2 antigen were expressed: the entire ORF2 (1–660 aa), its C-terminal portion (394–660 aa), and five 100–120 aa long ORF2 peptides (named peptide 1 to 5) were expressed in *E. coli*, while an N and C-terminal truncated ORF2 (112–608 aa) was produced in the baculovirus expression system. The HEV ORF3 coding region was also expressed in *E. coli*. The expressions in *E. coli* and baculovirus were conducted by sub-cloning the coding region of interest into pQE30 (Qiagen, Hilden, Germany) and pFastBac (Life Technologies, Carlsbad, CA, USA) vectors, respectively. The production of the proteins was described previously [21]. The proteins expressed in *E. coli* were purified by His-tag at the N-terminal position using a Ni-NTA resin according to the manufacturer’s instructions (Qiagen Hilden, Germany). All the proteins expressed were checked by SDS-PAGE and indirect ELISA. The entire (1–660 aa) and the truncated ORF2 (112–608 aa) were analyzed by western blotting using serum from an experimentally infected pig. The western blotting is described below.

### 2.3. Production and Characterization of Monoclonal Antibodies

Two BALB/c mice were immunised with 2–3 doses of 80 µg per dose of the purified ORF2 1–660 aa protein. The fusion of mouse splenocytes and NS0 myeloma cells was conducted according to standardised procedures within the laboratory. Hybridomas supernatants were screened by indirect ELISA against the homologous antigen ORF2 (1–660 aa). The isotype of the MAbs was determined using a commercial ELISA kit (BD-Pharmingen, Franklin Lakes, NJ, USA) following the manufacturer’s instructions.

MAbs were characterized by western blotting and mapped by indirect ELISA assays against all the recombinant constructs expressed by *E. coli*. Their reactivity with the recombinant ORF2 (112–608 aa) expressed by baculovirus was also evaluated by immunofluorescence on Sf9 infected cells. Ten hybridomas (1A2, 3E9, 2C7, 1E2, 2G8, 1D4, 1D10, 3C3, 3C4, and 4E12) recognizing different portions of the ORF2 antigen were selected and cloned by limiting dilution to ensure monoclonality and stability. The selected MAbs were purified by Protein A affinity chromatography and conjugated with horseradish peroxidase (HRP-Sigma/Merck, Darmstadt, Germany) [22] to be evaluated in trapping, competitive, and sandwich ELISA assays.

### 2.4. Swine Sera

Four positive control sera (named 1331, 1437, 1442, and 1449) collected 28 days after an experimental infection and two negative sera from specific pathogen-free pigs were used as controls in all the assays. A total of 414 swine sera collected from various Italian herds and previously classified as positive (178) and negative (236) by Pezzoni et al. (2014) were used to evaluate the immunogenicity of the ORF3. A total of 200 sera of this collection classified as positive (116) and negative (84) were used to better draw ORF2 antigenicity by using five peptides encompassing ORF2.

### 2.5. Western-Blotting

Western blotting (WB) was performed using a standard protocol. Briefly, recombinant proteins denatured in SDS and β-mercaptoethanol were separated on precast polyacrylamide gels at 4–12% gradient concentrations (Novex^®^ Tris-Glycine, Thermo Scientific, Waltham, MA, United States) and transferred to nitrocellulose filters [23]. MAb tissue cultures were diluted 1/5 in a phosphate saline buffer at pH 7.2 containing 1% *w/v* of bovine albumin and 0.05% *w*/*w* of Tween 20. MAb binding was detected by incubation with HRP-labelled rabbit anti-mouse IgG and the chemiluminescent substrate Amersham ECL plus (GE healthcare, Chicago, IL, USA). Swine sera were diluted 1/20 in the phosphate buffer described above. Antibody binding was detected by incubation with an HRP-conjugated anti-swine IgG monoclonal antibody using a chemiluminescent substrate as described above.

### 2.6. Indirect ELISA

An Indirect ELISA was conducted to map the MAbs and study the immunological profile of five ORF2 peptides and the ORF3 antigen with swine field serum samples. Optimal coating dilutions of all the antigens used for MAbs mapping were previously determined using the serum from immunized mice, ORF2 peptides and ORF3 antigen were also pre-titrated using known HEV positive and negative pig sera. The purified antigens expressed in *E. coli* were adsorbed in a carbonate-bicarbonate buffer onto ELISA microplates (Nunc Maxisorp, Thermo Scientific, Waltham, MA, USA) by overnight incubation at 4 °C. After washing, MAbs (hybridomas cultures supernatants 1/2 diluted) or serum samples (1/100 dilution) were added and their reaction was monitored after washes by HRP-conjugated anti-mouse or anti-swine IgG at the appropriate dilution. The diluting buffer was PBS with 0.05% Tween 20 and 1% yeast extract; 50 µL/well of reagent and 1 h of incubation at 37 °C were the conditions adopted for both steps. Then, the substrate OPD (o-Phenylenediamine 0.5 mg/mL in phosphate-citrate buffer pH 5.6 supplemented with 0.02% H_2_O_2_) was added for 10 min at room temperature. The reaction was stopped with 50 µL of 1M H_2_SO_4_ and absorbance values were read at 492 nm.

When serum samples were tested, sera were delivered in duplicate wells, one coated with the antigen and one conditioned with carbonate buffer alone as a negative antigen control, and a 1% *v/v* of negative *E. coli* extract was added to the serum dilution buffer to block non-specific reactions when *E. coli* expressed proteins were used. For each tested and control serum, the net Optical Density (OD) value was calculated by subtracting the OD value of the well without antigens from the OD of the well containing the antigen. The variation between plates and experiments was normalized by calculation of the percentage of positivity to a positive control serum present in each plate according to the formula: (net OD sample/net OD positive control) × 100.

### 2.7. MAb Competitive Binding ELISA

The assay was conducted in ORF2 (1–660 aa) coated microplates (Nunc Maxisorp, Thermo Scientific, Waltham, MA, USA). Fifty microliters of hybridoma culture supernatants were incubated sequentially diluted from 1/2, followed by the addition of 25 μL of HRP-conjugated MAb. After washing, the colorimetric reaction was developed as previously described. Results were expressed as the percentage inhibition of the binding of HRP-conjugated MAb. The amount of binding obtained in the absence of an unlabeled antibody (competitor MAb) was set at 100% for each HRP-conjugated MAb. The optimal dilution of the ORF2 (1–660 aa) and each conjugated MAb was predetermined in a preliminary checkerboard titration to give an absorbance value of 1–1.5 OD

### 2.8. Sandwich ELISA

ELISA plates (Nunc Maxisorp, Thermo Scientific, Waltham, MA, USA) were coated overnight at 4 °C with the capture MAb (10 µg/mL partially purified in a carbonate-bicarbonate buffer) and washed. Fifty microliters of each antigen sequentially diluted were then distributed and plates were incubated for 1 h at 37 °C. After washing, 50 µL of predetermined optimal dilution of HRP-conjugated MAb were added and incubated as before. After a final washing cycle, the reaction was developed with OPD as previously described.

### 2.9. Trapping ELISA

A trapping ELISA was used to evaluate the reactivity of positive serum samples from experimental infection against 3 recombinant ORF2 antigens variably captured by each of the selected MAbs. ELISA microplates (Nunc Maxisorp, Thermo Scientific, Waltham, MA, USA) were coated with each partially purified MAb at 10 µg/mL in a carbonate-bicarbonate buffer. The 3 recombinant antigens (ORF2 1–660 aa, ORF2 112–608 aa, and ORF2 394–660 aa) were added at sequential dilution to each coated MAb and incubated. Then, HEV positive and negative sera diluted 1:100 were distributed, followed by a home-made peroxidase-conjugated anti-swine IgG MAb; the reaction was finally developed using OPD. Each step was separated by washing cycles with PBS-Tween 20; 50 µL per well of reagent were used; the diluting buffer was PBS (pH 7.4) with 0.05% Tween 20 supplemented with 1% yeast extract and 1% mouse serum. As described for indirect ELISA, sera were delivered in duplicate wells: one coated with the antigen captured by the MAb and one with the MAb alone as the negative control. A 1% *v/v* of negative *E. coli* extract was also added to serum dilution buffer to block non-specific reactions when *E. coli* expressed proteins were used. For each tested and control serum, the net OD value was calculated as previously described.

### 2.10. Sera Competitive ELISA

This competitive ELISA was used to evaluate the capability of HEV positive sera to inhibit the binding of selected MAbs to the corresponding target epitopes. The purified antigens ORF2 (1–660 aa) and ORF2 (394–660 aa) were directly adsorbed onto the wells of the NUNC Maxisorp plates (Thermo Scientific, Waltham, MA, USA), while the baculovirus unpurified ORF2 (112–608 aa) was immune-captured by 4E12 MAb adsorbed at 10 µg/mL. Antigens and MAb coating was achieved by overnight incubation at 4 °C with reagents diluted in a carbonate-bicarbonate buffer. Fifty microliters of positive and negative serum samples were incubated at sequential two-fold dilutions starting from 1/4 and incubated for 1 h at 37 °C. Without washes, 25 μL of HRP-conjugated MAbs were added and incubated for 1 h further at 37 °C. After washing, the colorimetric reaction was developed as described above. Results were expressed in OD. The optimal concentration of the antigens and the conjugated MAbs were obtained by a checkerboard titration to obtain an OD of 1–1.5.

## 3. Results

### 3.1. Production of Recombinant Proteins and Peptides

**Figure 1:**
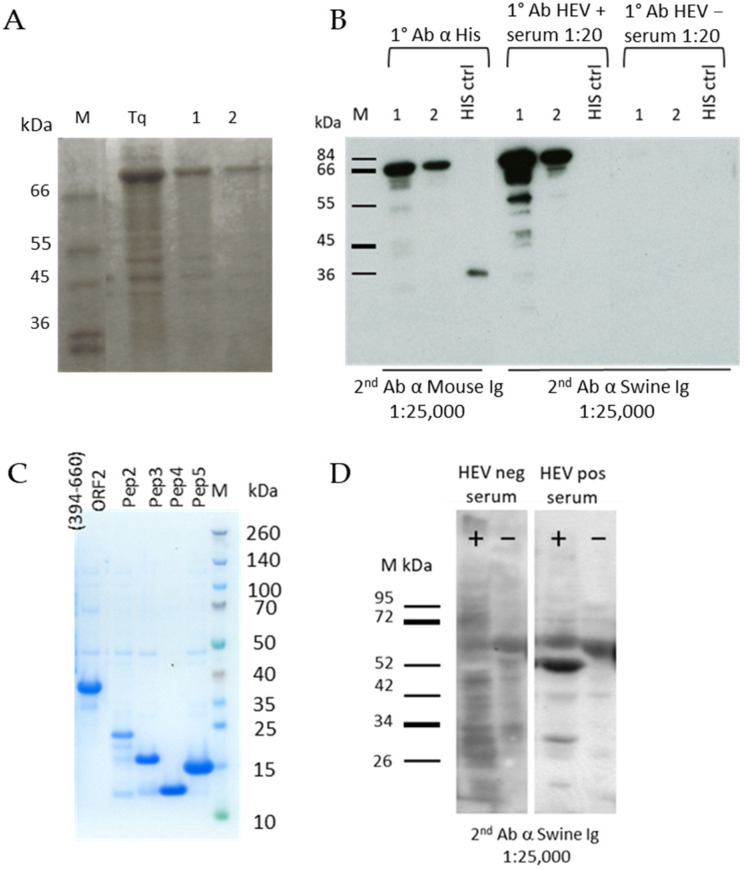
(**A**) SDS page of purified ORF2 (1–660 aa) (72 kDa) at three different concentrations: (Tq) 1 µg, (1) 500 ng, and (2) 250 ng. M: molecular weight marker. (**B**) Western blotting of the entire ORF2 (1–660 aa) at 500 ng (1) and 250 ng (2) with an anti-Histidine antibody and swine HEV positive and negative sera. A Histidine positive control (His ctrl) was added. Sera were diluted 1:20. (**C**) SDS page of purified ORF2 (394–660 aa) and the four peptides, namely peptide 2 (19.8 kDa), peptide 3 (13.9 kDa), peptide 4 (10.7 kDa), and peptide 5 (13.8 kDa). (**D**) Western blotting of ORF2 (112–608 aa) expressed in the baculovirus system. Lane +: crude cells lysate infected by baculovirus expressing ORF2 (112–608 aa) (53kDa), lane −: crude cells lysate infected by baculovirus used as negative control. Swine positive and negative sera were diluted 1:20.

The recombinant proteins expressed in *E. coli* were all extracted in denatured conditions and purified by Immobilized-Metal Affinity Chromatography (IMAC). The yield of the entire ORF2 (1–660 aa) antigen after purification was 1.17 mg from 100 mL of bacterial culture (Figure 1A). The protein was recognized in western blotting by an anti-Histidine MAb and by a positive serum from an experimentally infected pig at the expected molecular weight (72 kDa) (Figure 1B). The C-terminal one-third portion (394–660 aa) was expressed with a yield of 25 mg from 100 mL of bacterial culture and the correct molecular weight of 30.5 kDa was evidenced by SDS-PAGE (Figure 1C). Both the ORF2 (1–660 aa) and ORF2 (394–660 aa) were efficiently detected by HEV positive sera when directly adsorbed onto ELISA microplates (not shown). Among the ORF2 peptides, the first one (Pep1 102–272 aa) was poorly expressed and the other four were produced at about 1 mg yield from 100 mL of bacterial culture, their expression monitored by SDS-PAGE (Figure 1C). The recombinant ORF2 (112–608 aa) expressed by the baculovirus system was evidenced in the infected cells culture medium and cells lysate by western blotting (Figure 1D) with an HEV positive serum. The molecular weight corresponded to that as expected (53 kDa).

**Figure 2:**
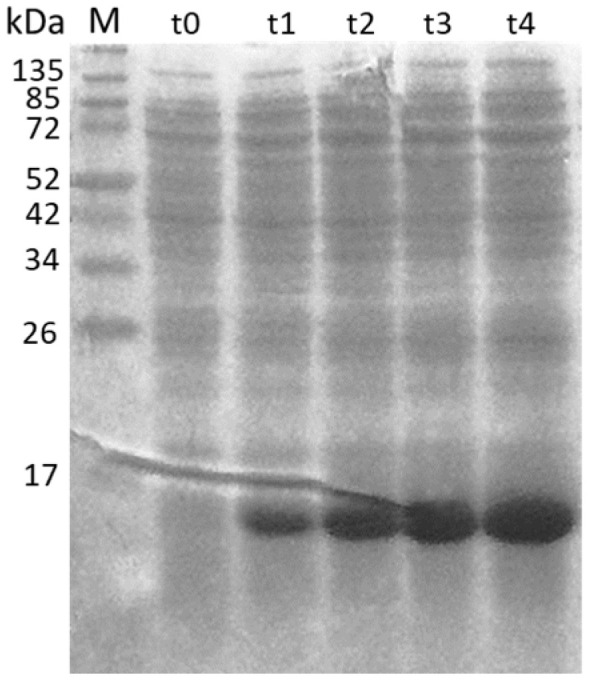
SDS page of the time course of the expression of ORF3 (13.6 kDa) recombinant protein. The expression was evaluated at different time points (t) corresponding to hours post-induction. M: molecular weight marker.

The ORF3 was confirmed to be expressed by SDS-PAGE at the correct molecular weight of 13.6 kDa (Figure 2).

### 3.2. Evaluation of ORF2 Antigenicity with MAbs

Thirty-seven MAbs were selected for their specific reactivity against the *E. coli* expressed ORF2 (1–660 aa) in indirect-ELISA; the MAbs obtained were isotyped as reported in Table 1. All MAbs were analyzed in western blotting against the entire ORF2 (1–660 aa) antigen: 28 of them demonstrated a strong signal and three reacted faintly with a band at the expected molecular weight of 72 kDa, while six MAbs did not produce any signal. Thirty-four MAbs exhibited reactivity against Sf9 cells infected with recombinant baculovirus expressing ORF2 (112–608 aa) in immune-fluorescence (Table 1).

**Figure 3:**
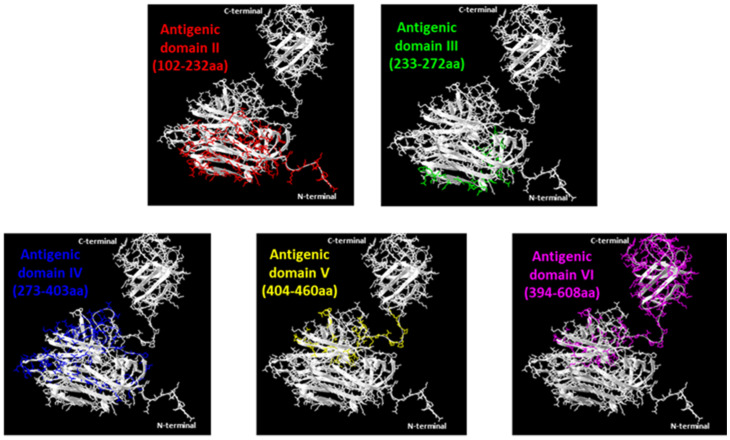
Ribbon diagrams of crystal structures of ORF2 capsid protein (118–606 aa) (PDB accession code: 2ZZZQ). Five of the six domains identified are indicated with different colors. The pictures were obtained by SwissPdb Viewer [24].

**Figure 4:**
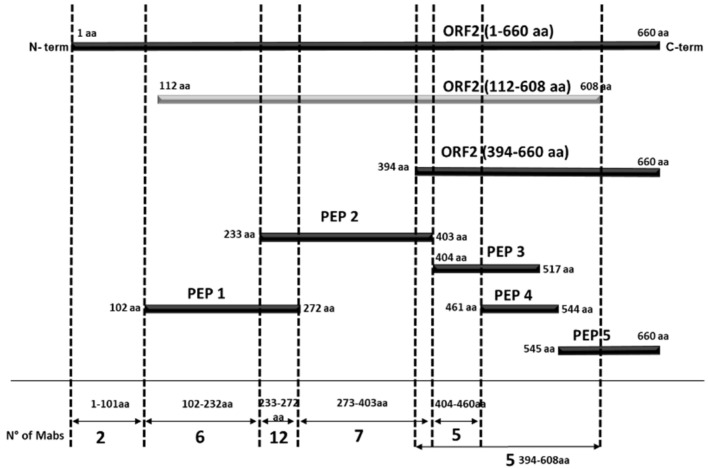
Schematic representation of the ORF2 recombinant proteins/peptides produced. The numbers of MAbs that recognize various portions of ORF2 are presented at the bottom of the diagram. The proteins expressed in *E. coli* are highlighted in black, while the ORF2 (112–608 aa) expressed in baculovirus is highlighted in light grey.

The MAbs were mapped based on their pattern of reactivity with *E. coli* expressed recombinant antigens encompassing different portions of the ORF2 protein in indirect ELISA assays (Table 1). When two overlapping regions were recognized, we assumed that the binding area is included in the common amino acid sequence of the two overlapping portions. Differently, when only one of two overlapping peptides demonstrated reactivity, the target sequence was assigned to the non-overlapping segment. By MAbs mapping, we identified six contiguous domains along the ORF2 protein (Table 1, Figure 3 and Figure 4).

Two MAbs (1A2 and 3E9) that recognized only the entire ORF2 but none of the portions spanning from aa position 102 to the end probably reacted with the N-terminal ORF2 portion 1–101 aa representing domain I, while one MAb (5G1) should map in the short region spanning between 102 and 111 aa, which is the unique sequence present in peptide 1 and not in other ORF2 sub-portions. Five further MAbs identifying domain II were mapped between 112–232 aa (i.e., the region common to the ORF2 112–608 aa and peptide 1 but absent in any following protein portions). Twelve MAbs recognized domain III, mapping in the common sequence of peptide 1 and 2, therefore between 233–272 aa positions. Domain IV is the target region of another seven MAbs, mapping in the distinctive sequence of peptide 2, between 273–403 aa positions. Five MAbs identifying domain V exhibited reactivity in the segment 404–460 aa (unique in peptide 3). Finally, five MAbs recognized a truncated ORF2 encompassing the C-terminal 394–660 aa region but the target epitope was not detected in any of its three sub-fragments examined (Table 1, Figure 4). Domains II to VI were visualized in the 3D structure of the ORF2 capsid protein (118–606 aa) obtained by SwissPdb Viewer [24] (Figure 3).

Ten hybridomas (1A2, 3E9, 2C7, 1E2, 2G8, 1D4, 1D10, 3C3, 3C4, and 4E12) recognizing different portions of the ORF2 antigen were selected for cloning and further culture amplification to be used as partially purified or as peroxidase conjugated in further ELISA assays.

### 3.3. Epitopes Differentiation by MAbs Reciprocal Competition

The ten HRP-conjugated MAbs were used to further characterize all 37 hybridomas by analyzing the reciprocal competition in a MAbs-competitive ELISA. Each MAb in the format of sequential dilutions of a hybridoma culture medium that included the saturating concentration was incubated in ELISA plates coated with ORF2 expressed in *E. coli*, followed by the addition of a conjugated MAb. If during the first incubation the unlabeled MAb blocked the subsequent binding of the conjugated MAb, we could assume that the MAbs recognized a similar or at least an overlapping epitope. The results evidenced that MAbs 1A2 and 3E9 exhibited the same profile and both were inhibited by MAb 5G1 from binding to the ORF2 antigen, confirming that all the three MAbs mapping in the same ORF2 region (1–111 aa) recognized the same or an overlapping epitope. Nine out of twelve MAbs binding within to the 233–272 aa ORF2 region (domain III) were clustered in same group as they inhibited the binding of both HRP-conjugated 2C7 and 1E2, demonstrating the same reactivity profile. No one MAb blocked the binding of HRP-conjugated 1D4, suggesting that it recognizes a further distinct epitope within the domain III. Four out of the seven MAbs mapped in the domain IV (ORF2 273–403 aa region) competed with HRP-conjugated 1D10, thus identifying a unique epitope, while no one MAb competed with the 2G8 conjugate that therefore maps to a distinct target epitope within domain IV. In domain V, MAb 3C3 inhibited the binding of HRP-conjugated 3C4 and vice versa, and both were blocked by MAb 2C12. Finally, the group of five MAbs that recognized a truncated ORF2 encompassing the C-terminal 394–660 aa region but were not mapped in any of the sub-fragments demonstrated a similar reactivity profile, all competing with HRP-conjugated 4E12.

### 3.4. Evaluation of the Conformational Properties of the Different ORF2 Recombinant Antigens Using Sandwich ELISA with MAbs

**Figure 5:**
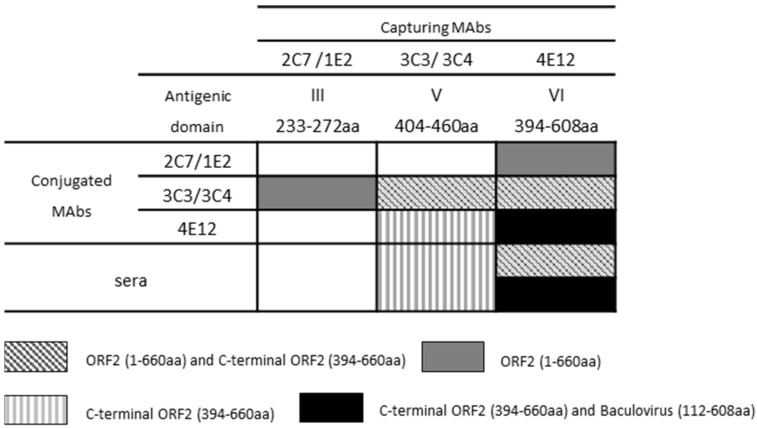
Schematic representation of the ability of the selected MAbs to capture the various recombinant proteins and expose them to the recognition of conjugated MAbs or four HEV-positive swine sera from experimental infection in sandwich and trapping ELISA, respectively.

To evaluate the conformational characteristics of the recombinant antigens, all the selected MAbs were used in combination as capture and HRP-conjugated for the detection of ORF2 (1–660 aa), ORF2 (112–608 aa), and ORF2 (394–660 aa) (Figure 5). The ORF2 (1–660 aa) was efficiently detected by 3C3 and 3C4 HRP-conjugated MAbs when captured by both 2C7 and 1E2 MAbs (heterologous MAb combination). The MAbs 3C3 and 3C4 could capture and detect the antigen even in homologous combinations in the same sandwich reaction. MAb 4E12 used as the catcher of ORF2 (1–660 aa) allowed 2C7, 1E2, 3C3, and 3C4 to detect the recombinant antigen; no reactivity was observed with any of the other MAbs combinations. The ORF2 expressed in baculovirus (112–608 aa) demonstrated to be efficiently captured and detected only by 4E12 MAb, while the C-terminal ORF2 (394–660 aa) was detected by 3C3, 3C4, and 4E12 in all possible combinations.

### 3.5. Analysis of Immunogenicity of the Different ORF2 Recombinant Antigens Using Trapping ELISA

A trapping ELISA was conducted to evaluate the ability of each of the ten selected MAbs to capture the ORF2 recombinant antigens exposing immunogenic epitopes to the recognition of a representative panel of four HEV positive sera from an experimental infection in pigs (Figure 5). The ORF2 (1–660 aa) captured by MAb 4E12 was efficiently detected by HEV positive sera. Only MAb 4E12 captured the ORF2 (112–608 aa) expressed in baculovirus and exposed it to the recognition of positive sera. The C-terminal portion of the ORF2 (394–660 aa) was efficiently captured by MAbs 3C3, 3C4, and 4E12 with the exposition of immunogenic epitopes. Only MAbs belonging to the antigenic domain V and VI demonstrated to efficiently capture the recombinant proteins in trapping ELISA.

### 3.6. Evaluation of MAbs Target Epitopes Immunogenicity Using Competitive ELISA

**Figure 6:**
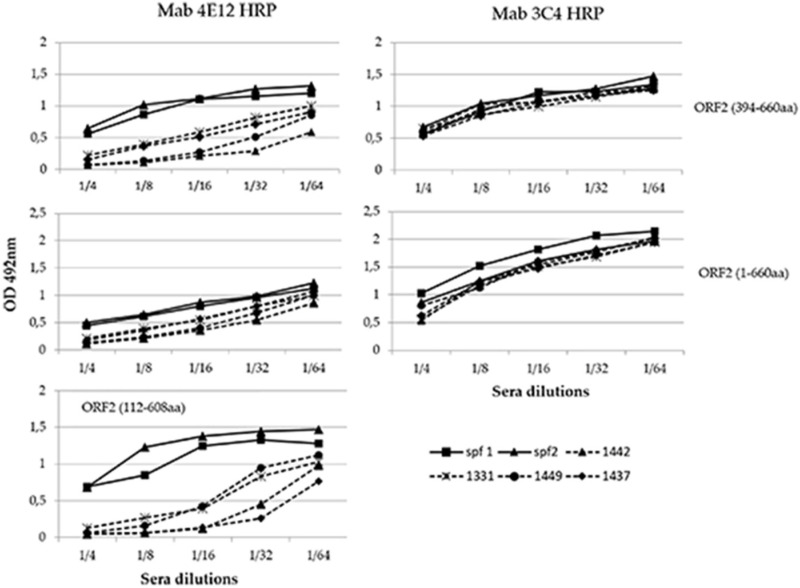
Competition profile of four positive sera (1331, 1437, 1442, and 1449) and two negative controls (spf1 and spf2) with MAb 4E12 and 3C4.

This experiment was conducted to evaluate the capability of known HEV positive sera to inhibit the binding of the selected MAbs to the antigen and precisely to determine if the epitopes recognized by each MAb were immunogenic. Sera from pigs experimentally infected with HEV and from specific HEV-negative pigs were tested in a competitive ELISA assay with each HRP-conjugated MAb. Among the selected MAbs, only 4E12 demonstrated to compete with the swine HEV positive sera (Figure 6). A clear competition of 4E12 was evident particularly with baculovirus-expressed ORF2 (112–608 aa) and in a lesser extent with C-terminal ORF2 (394–660 aa), while it was minimal with ORF2 (1–660 aa). The MAb 3C4 as well as the others conjugated MAbs did not compete with the positive sera that exhibited the same profile as the negative sera. The baculovirus-expressed ORF2 (112–608) was evaluated only in association with the conjugated MAb 4E12 because, as evidenced by sandwich ELISA experiments (see above), no other conjugated MAb recognized this antigen when captured by 4E12.

### 3.7. Evaluation of Immunogenicity of ORF2 Peptides and ORF3 Using Field Sera

**Figure 7:**
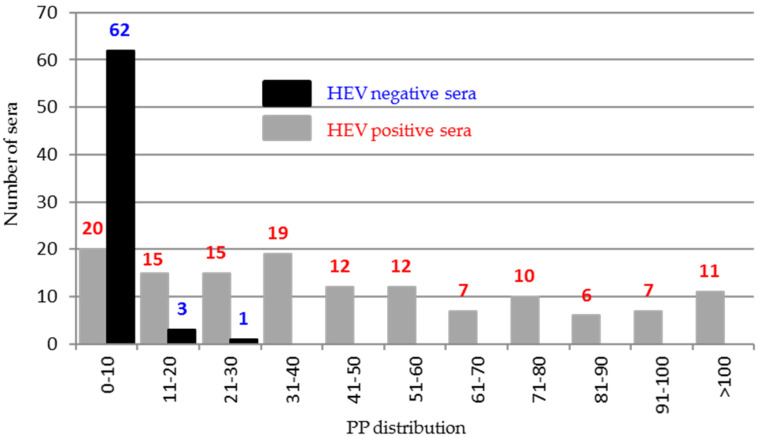
Distribution of the percentage of positivity (PP) of the 200 HEV positive and negative sera tested in indirect ELISA against ORF2 peptide 5 (545–660).

To better draw ORF2 immunogenicity, 200 sera classified as negative (66) or positive (134) by combined results of previous ELISAs reported by Pezzoni et al. (2014) were tested against five recombinant peptides encompassing the entire protein using an indirect ELISA. Among the five peptides, only peptide 5 (544–660 aa) was found to differentiate positive and negative HEV sera. The majority of negative sera (62/66, 93%) clustered in the PP value range of 0–10% and in contrast 114/134 (85%) positive sera presented PP values higher than 10% (Figure 7).

**Figure 8:**
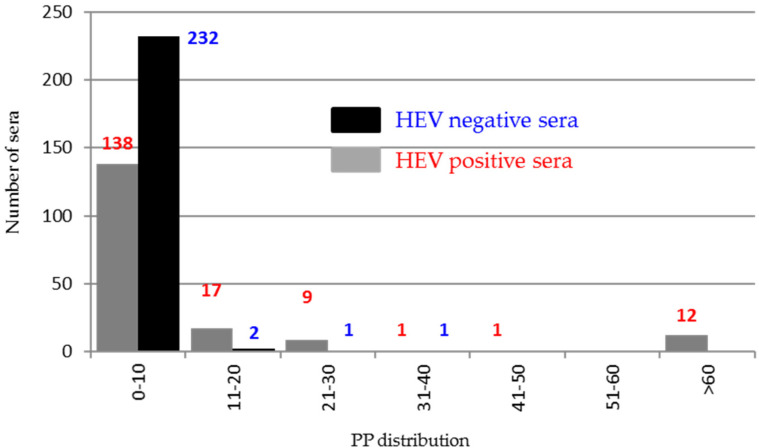
Distribution of the percentage of positivity (PP) of the 414 HEV positive and negative sera tested in indirect ELISA against the ORF3 recombinant protein.

Using ORF3 as an antigen, the evaluation of 414 sera classified as negative (236) and positive (178) [21] demonstrated that 99% of negative sera gave PP values in the range of 0–20%, while only 22/178 (12%) of positive sera were clearly reactive with PP values higher than 20% (Figure 8).

## 4. Discussion

In this work, the antigenic and immunogenic properties of ORF2 and ORF3 antigens from HEV genotype III were studied using MAbs and swine sera. Although the ORF2 antigen was extensively studied using MAbs in humans [17,25,26] to develop an effective vaccine [27], only a few researchers evaluated its antigenic characteristics in swine [28,29]. Conversely, the role of ORF3 giving rise to an immune response has not been completely investigated.

The entire ORF2 encoded protein (1–660 aa) of HEV genotype III from swine was expressed as a recombinant protein in *E. coli* and used to immunize two mice obtaining a panel of MAbs covering the entire antigen. A series of other ORF2 recombinant antigens were produced both to precisely map the MAbs and study the immunogenic properties of the ORF2 protein using HEV positive swine sera. In addition to the entire ORF2 (1–660 aa), the expressed recombinant proteins included a truncated ORF2 (112–608 aa) expressed in the baculovirus system, where it seems to self-assemble in virus-like particles [30], and a truncated ORF2 (394–660 aa) that previous studies described as an immune-dominant region [12]. Another five peptides were also produced in *E. coli* as ORF2 sub-fragments. All 37 MAbs obtained proved to recognize the entire ORF2 (1–660 aa) in indirect ELISA, 34 reacted in immunofluorescence experiments against the baculovirus expressed ORF2 (112–608 aa), and 28 detected in western blotting the entire ORF2, proving to recognize linear epitopes.

According to their reactivity against the recombinant antigens, the MAbs were grouped and revealed six antigenic regions on the ORF2 protein 1–608 aa. Within each region, MAbs were sub-grouped in clusters based on the results in reciprocal competitive ELISA.

The region encompassing 1-101 aa includes the epitope target of the two MAbs 1A2 and 2E9 that competed reciprocally. In addition, despite mapping in the second antigenic region of 102–231 aa, MAb 5G1 is suggested to recognize a close or overlapping epitope given its capability to compete with both 1A2 and 2E9. The second antigenic region includes five further MAbs. Among the twelve MAbs mapped in the third antigenic region (233–272 aa), nine seem to recognize the same linear epitope as all displayed a similar competition profile: this cluster includes MAbs 2C7 and 1E2. Of the three remaining MAbs, 1D4 identifies a distinct epitope as no other Mab inhibited its binding, while 2D6 and 3H7 map to different epitopes. In the fourth antigenic region, MAb 1D10 clusters with four other MAbs, while MAbs 2G8 and 3E7, which exhibited individually different competition profiles, likely identify two further independent epitopes. A total of five MAbs exhibited the same reaction profile and are grouped in the fifth antigenic region. Among these, MAbs 3C3, 3C4, and 2C12 competed with each other, demonstrating to recognize the same epitope. The five MAbs belonging to the sixth group recognized the truncated ORF2 (394–660 aa) but failed to react with the sub-fragments encompassing this region. MAbs belonging to this group represented by 4E12 were supposed to recognize the same or overlapping epitopes, as all competed against 4E12 for the binding to ORF2.

Sandwich ELISA assays performed with MAbs as capture and detection reagents can also be useful to characterize the antigenic structure of proteins and more complex antigens, considering that such assays can efficiently detect the relevant antigens when the capture and the detector antibody map to different epitopes or when they recognize the same epitope providing it is present in multiple copies. The sandwich ELISA assay illustrated that out of ten MAbs reacting with different domains, only MAbs of domains III, V, and VI, namely 2C7/1E2, 3C3/3C4, and 4E12, can capture the full-length ORF2 (1–660 aa) and expose it to reciprocal recognition; the target epitopes of these MAbs are probably externally exposed in the full-length protein expressed in *E. coli*. However, not all the combinations of capture and detector MAbs worked efficiently, presumably depending on different ways of exposure and orientation of the involved epitopes. Unexpectedly, the ORF2 (1–660 aa) antigen was reactive with the homologous combination of MAbs 3C3 and 3C4, indicating a homodimer formation or aggregation of ORF2 (1–660 aa) with the consequent presence of multiple copies of the target epitope. Despite the observation that the MAbs captured the full-length ORF2, only MAb 4E12 was able to expose it for a favourable recognition of positive sera.

A previous study on ORF2 expressed in baculovirus demonstrated that the truncation of N-terminal 111 aa and C-terminal 52 aa improves the formation of VLPs [30]. In our study, ORF2 (112–608 aa) expressed in baculovirus confirmed to have a different folding in respect to the full-length ORF2 (1–660 aa) expressed in *E. coli*. Not one of the MAbs apart from 4E12 was able to catch this antigen and expose it to the recognition of the positive sera. Moreover, 4E12 bound and detected the baculovirus expressed ORF2 (112–608 aa) in sandwich ELISA experiments when used as both catcher and tracer in the same reaction, confirming the presence of multiple 4E12 epitopes due to the self-assembling of this antigen.

The C-terminal portion of ORF2 (394–660 aa) was regarded in many studies as the ORF2 portion responsible for the immune response against HEV and involved in protein dimerization [30,31,32]. Furthermore, the region encompassing 455–602 aa has been proved in a structural study to correspond to the protruding domain [31,33]. Our study confirmed ORF2 (394–660 aa) dimerization as all the tested MAbs mapped in this region, 3C3/3C2 and 4E12, were able to capture and detect the antigen in the same homologous sandwich reaction. These MAbs could also efficiently expose the captured antigen to the recognition of HEV positive sera.

The MAb 4E12 was the only one among all the ten selected and evaluated that competed with the swine HEV positive sera, confirming to recognize an immunogenic epitope exposed on all the three main recombinant antigens expressed. While MAb 4E12 reacted in indirect ELISA with the C-terminal portion of ORF2 (394–660 aa), we were not able to sub-map its epitope more precisely using shorter peptides. However, its absence of reactivity in western blotting suggests that MAb 4E12 likely recognizes a partial conformational epitope exposed by all the ORF2 proteins produced.

The indirect ELISA using positive and negative swine sera and ORF2 peptides confirmed the presence of an immunogenic region in the portion encompassing 544–660 aa. Conversely, only 12% of swine HEV positive sera demonstrated to react against ORF3 antigen, indicating a very low antigenicity of the ORF3 protein in pigs.

Overall, the results confirmed the region from 394 aa to 608 aa of ORF2 protein, corresponding to the fifth and sixth antigenic region targeted by MAbs as the most immunogenic, harboring both conformational and linear epitopes. The other antigenic regions denoted by MAbs could correspond to internal regions in the assembled capsid as illustrated by structural studies [33].

In this study, we obtained and mapped a panel of MAb that will be useful tools for further investigations on HEV biology. In addition, MAb 4E12 has the potential for diagnostic exploitation. Our results support previous studies regarding immunogenic regions of the ORF2 capsid protein, providing further information for vaccine development.

## Figures and Tables

**Table 1:** List and features of the monoclonal antibodies produced. The response of each MAbs to different portions of protein in western blotting. Immunofluorescence and ELISA is indicated by +, weak, and -; n.d. is defined as no-detected. Cloned MAbs are shown in bold. Lines colored in grey and encircled by the dashed line represent antibodies that were reciprocally reactive in competitive ELISA. A total of six immune domains were identified on ORF2 based on MAbs reactivity.

MAbs	Isotype	WB ORF2	ELISA ORF2 (1–660 aa)	IF ORF2 (112–608 aa)	ELISA						Domain
					ORF2 (394–660 aa)	Peptide 1 (102–272 aa)	Peptide 2 (233–403 aa)	Peptide 3 (404–517 aa)	Peptide 4 (461–544 aa)	Peptide 5 (545–660 aa)	
**1A2**	**IgG1**	**n.d.**	**+**	**-**	-	-	-	-	-	-	**I** **(1–101 aa)**
**3E9**	IgG1	+	+	-	-	-	-	-	-	-	
5G1	IgG1/IgG3/IgG2	+	+	-	-	+	-	-	-	-	**II** **(102–232 aa)**
5G5	IgG1/IgG3/IgG2	+	+	+	-	+	-	-	-	-	
5H9	IgG1	+	+	+	-	+	-	-	-	-	
1G3	IgG1	+	+	+	-	+	-	-	-	-	
2B3	IgG3	+	+	+	-	+	-	-	-	-	
4G12	IgG1	+	+	+	-	+	-	-	-	-	
**2C7**	IgG2a	+	+	+	-	+	+	-	-	-	**III** **(233–272 aa)**
**1E2**	IgG1	+	+	+	-	+	+	-	-	-	
5F8	IgG3/IgM	+	+	+	-	+	+	-	-	-	
4E6	IgG2a	+	+	+	-	+	+	-	-	-	
2G10	IgG3	+	+	+	-	+	+	-	-	-	
2F6	IgG3	+	+	+	-	+	+	-	-	-	
2D7	IgG1	+	+	+	-	+	+	-	-	-	
5F7	IgG3	+	+	weak	-	+	+	-	-	-	
2C6	IgG2a	+	+	weak	-	+	+	-	-	-	
2D6	IgG2a/IgM	+	+	weak	-	+	+	-	-	-	
3H7	IgG2a	+	+	+	-	+	+	-	-	-	
**1D4**	n.d.	weak	+	+	-	+	+	-	-	-	
**2G8**	IgG1	n.d.	+	+	-	-	+	-	-	-	**IV** **(273–403 aa)**
**1D10**	IgG1	weak	+	+	-	-	+	-	-	-	
1G4	IgG1	+	+	+	-	-	+	-	-	-	
5E9	IgG1	+	+	+	-	-	+	-	-	-	
5C10	IgG1	+	+	+	-	-	+	-	-	-	
1H9	IgG1	n.d.	+	+	-	-	+	-	-	-	
3E7	IgG1	weak	+	+	-	-	+	-	-	-	
3D9	IgG1/IgG3/IgG2b	+	+	+	+	-	-	+	-	-	**V** **(404–460 aa)**
**3C3**	IgG2b	+	+	+	+	-	-	+	-	-	
**3C4**	IgG2b	+	+	+	+	-	-	+	-	-	
2C12	IgG2a	+	+	+	+	-	-	+	-	-	
3A8	IgG1	n.d.	+	+	+	-	-	+	-	-	
**4E12**	IgG1	n.d.	+	+	+	-	-	-	-	-	**VI** **(394–608 aa)**
5D10	IgG1	+	+	+	+	-	-	-	-	-	
4G6	IgG1	n.d.	+	+	+	-	-	-	-	-	
5G9	IgG1	+	+	+	+	-	-	-	-	-	
5A8	IgG1	+	+	+	+	-	-	-	-	-	

## Data Availability

The data presented in this study is available within the article.
